# Gait Analysis Accuracy Difference with Different Dimensions of Flexible Capacitance Sensors

**DOI:** 10.3390/s21165299

**Published:** 2021-08-05

**Authors:** DongWoo Nam, Bummo Ahn

**Affiliations:** 1 Robot Division, Korea Institute of Industry Technology, Ansan 15588, Korea; sdrcat@kitech.re.kr; 2 School of Korea Institute of Industry Technology, Robotics and Virtual Engineering, University of Science and Technology, Ansan 15588, Korea

**Keywords:** gait analysis, flexible capacitance sensor, dimension, ankle angle

## Abstract

Stroke causes neurological pathologies, including gait pathologies, which are diagnosed by gait analysis. However, existing gait analysis devices are difficult to use in situ or are disrupted by external conditions. To overcome these drawbacks, a flexible capacitance sensor was developed in this study. To date, a performance comparison of flexible sensors with different dimensions has not been carried out. The aim of this study was to provide optimized sensor dimension information for gait analysis. To accomplish this, sensors with seven different dimensions were fabricated. The dimensions of the sensors were based on the average body size and movement range of 20- to 59-year-old adults. The sensors were characterized by 100 oscillations. The minimum hysteresis error was 8%. After that, four subjects were equipped with the sensor and walked on a treadmill at a speed of 3.6 km/h. All walking processes were filmed at 50 fps and analyzed in Kinovea. The RMS error was calculated using the same frame rate of the video and the sampling rate of the signal from the sensor. The smallest RMS error between the sensor data and the ankle angle was 3.13° using the 49 × 8 mm sensor. In this study, we confirm the dimensions of the sensor with the highest gait analysis accuracy; therefore, the results can be used to make decisions regarding sensor dimensions.

## 1. Introduction

Stroke is one of the most frequently occurring neural diseases worldwide. For instance, in the United States, 800,000 people experience a stroke every year [1]. After a stroke, most patients experience a neurological pathology resulting from the loss of the neural pathway in the spinal cord [2]. Drop foot is a typical example of a pathologic gait caused by a spinal cord disorder [3]. Patients with this pathologic gait cannot increase their ankle angle during walking; thus, they are at risk of falling. This type of neurological pathology has been difficult to diagnose in the past, but an objective analysis of gait disorders is currently possible with the development of diagnostic devices [4]. Gait analysis includes many methods, such as kinematics, kinetics, and electromyography [5]. Kinematics is a diagnostic method that measures the movement of human walking [5]. This method usually requires motion capture by infrared (IR) cameras, which is a widely used process because of its three-dimensional analysis and fast processing [4,5,6]. However, this process requires a large space for gait analysis between the cameras. Thus, this technique can only be performed in the laboratory and not in situ [4,6]. To address this drawback, many wearable systems for gait analysis have been used. Inertial measurement units (IMUs) are typical wearable instruments used in gait analysis [7]. Unlike IR cameras, these instruments can be used both in the laboratory and in situ [8]. Schauer et al. presented a study on joint angle measurements based on an IMU sensor [9]. They compared the results of normal and prosthetic joint ankle measurements. They also proposed a new algorithm for online use of the IMU, which has contributed to a more thorough gait analysis approach. However, the signal from the IMU sensor can be disturbed by unintended physical disturbances and the surrounding magnetic environments. Gracer et al. showed that IMU sensors had less accuracy than vision-based sensors because of the drawbacks mentioned in [10]. Moreover, the error associated with the IMU sensor increases with respect to time because of the continuous integration of acceleration [11]. These drawbacks can result in incorrect gait analysis results. The sensor material is also an important issue. Owing to the intricate structure of the human body and its movement, rigid sensing devices are difficult to use [12]. Moreover, because of their hard material, subjects feel uncomfortable during gait analysis [13] with commercial devices such as an IMU or a goniometer.

Flexible sensors have been used to detect human motion to address the drawbacks of existing gait detection devices [14]. For higher flexibility, silicone rubber has been frequently used in previous studies [15,16]. Due to its relatively high working range, continuous human movement can be captured [14,15,16,17,18]. The most famous type of flexible sensor is based on electric resistance to guarantee high accuracy [16]. For example, in [15], electric resistance-based flexible sensors were used to measure the movement of the lower limb joints. The results obtained with the sensors showed higher accuracy compared with the results obtained with an IR camera. However, electric resistance-based flexible sensors exhibit high hysteresis. Capacitance-based sensors have a lower accuracy than resistance-based sensors, but they have lower hysteresis [16]; thus, capacitance-based sensors are more reliable for measuring repeated movements, such as walking.

As flexible sensors can be attached [15,16,17,19,20], sewed [14,21], or directly fabricated [22] onto the clothes of a subject, they can be used as comfortable wearable devices. However, because of the structure of the human ankle, a directly fabricated sensor can be easily separated from the garment [22]. In addition, flexible sensors cannot be easily attached to the ankle when compared with other human joints, such as fingers [16], because of their curved structure. To overcome this problem, we propose a stable and simple structure with a metal ring to provide an easier and more stable connection in situ.

Many previous studies addressed the observation of human movements using flexible sensors. In [14], the authors considered the range of each lower limb joint to fabricate flexible sensors for each lower limb joint. The fabricated sensors were well matched for all subjects. A performance comparison between sensors with different dimensions, however, was not dealt with in [15]. In [16], a flexible sensor for observing finger movements was considered. Flexible sensors were used for each finger to measure the different movements of the hand and fingers. However, the dimensions of the subjects’ hands were not considered. Several studies have dealt with the topologies of flexible sensors to achieve high-quality monitoring of human movement [23,24]. In [23], flexible sensors placed at different positions were used to measure ankle joint kinematics in 10 healthy subjects. The authors presented the optimized placement of the sensors to measure the movement of the ankle with high accuracy, regardless of the subject’s body type. In [24], the authors measured shoulder kinematics with many flexible sensors. They found an optimized arrangement and number of sensors to measure the movement of the shoulder. Unfortunately, they did not consider the size of the sensor, which may change if different dimensioned sensors are used under the same conditions. If the morphology of the sensor is considered, the topologies can be approved and are more reliable.

**Figure 1:**
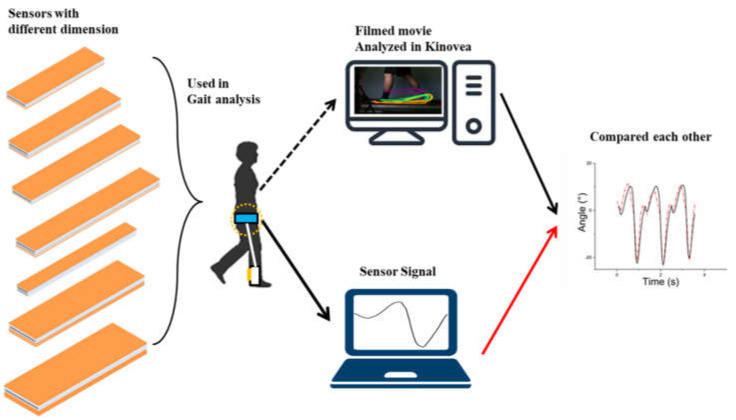
Main concept of this study. The subject walks on a treadmill with the sensor on the ankle. The walking procedure is filmed by a DSLR camera and analyzed on a PC through Kinovea. At the same time, the sensor signal is directly received by another computer. A total of seven procedures were carried out using sensors with different dimensions.

Therefore, in this study, a performance comparison of flexible sensors with different dimensions was performed. The movement of the ankle changed tremendously with a drop foot gait. Thus, we focused on the ankle movement in the sagittal plane. To determine the optimized dimensions of the flexible sensor, we fabricated seven sensors with different dimensions based on the average human body size and movement range. The sensors were characterized by repetitive tension using a linear actuator. After characterization, the sensors were equipped with an ankle brace and used to analyze ankle movement. Subjects walked on a treadmill at a speed of 3.6 km/h. To evaluate the accuracy of the sensor, all gait procedures were filmed and analyzed using a video analysis program (Kinovea, Boston, USA). Through this approach, the optimized dimensions of the flexible sensor were determined. Figure 1 shows the entire procedure used in this study as a simple diagram.

## 2. Materials and Methods

### 2.1. Basic Principle and Sensing System Overview

The capacitance of a wearable flexible sensor varies with the sectional area and distance, owing to the flexion or contraction of the sensor. The electric capacitance can be calculated using the following simple formula:

$$C=\varepsilon_{0}\varepsilon_{r}\frac{lw}{d}$$

**Figure 2:**
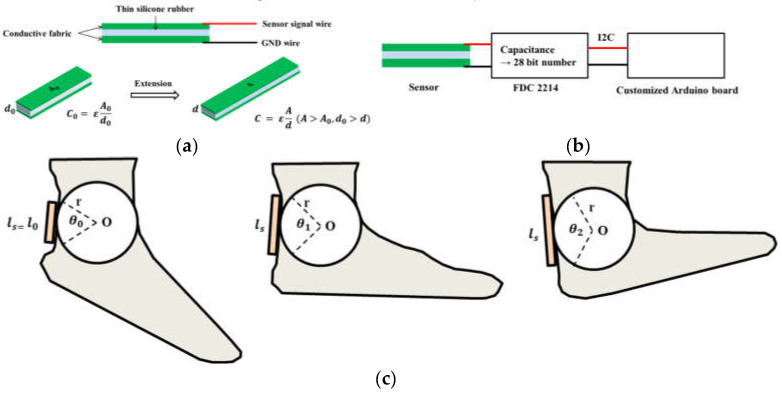
(**a**) Structure and principle of the flexible sensor. The sensor consists of two conductive fabric pieces as electrodes and a thin silicone rubber dielectric layer. A change in the length of the sensor causes a change in the capacitance level, and measuring the capacitance level is the operating principle of the sensor. (**b**) Entire circuit of the flexible sensor system. The FDC 2214 board changes the capacitance value to a 28 bit digital number. The Arduino board displays a digitized number. (**c**) Conversion of ankle angle to strain of the sensor. We assumed that the sensor is extended by the cylindrical behavior of the ankle angle.

In this equation, *l* and *w* are the length and width of the conductivity layer, respectively; $\varepsilon_{0}$ and $\varepsilon_{r}$ represent the vacuum permittivity ($\varepsilon_{0}=8.854\times 10^{-12}C/Vm$) and relative permittivity of silicone rubber, respectively ($\varepsilon_{r}=2.8\;C/Vm$ for Ecoflex 0030); and, finally, *d* denotes the distance between the electrode layers. By using this equation, the capacitance can be estimated. When both the conductive and electrode layers are flexed, the distance between each electrode layer decreases, and the area of the conductive layer expands because of the expansion of the length of the sensor. Thus, the capacitance increases. Using this theory, the body movement can be measured. Figure 2a shows the simple structure and principle of the sensor. The two green layers represent the electrodes. The gap between the two electrodes was filled with silicone rubber as the conductivity layer. Both sides of the electrodes were connected to a board with a capacitance-sensing module (Figure 2b).

The strain of the sensor should be converted to the ankle angle. The following equation is a simple model of strain–angle conversion. The detailed model is presented in Figure 2c.

$$l_{s}=l_{0}+r\sin(\theta_{0}+\Delta \theta )$$

where $l_{0}$ and $l_{s}$ denote the initial length and the length in the given situation of the sensor, respectively. As shown in Figure 2c, we assumed movement of the ankle as a cylindrical joint with a radius *r*. $\theta_{0}$ denotes the initial central angle of the ankle, and Δθ denotes the angle variation in the ankle joint.

### 2.2. Sensor Fabrication

To fabricate the sensor, a conductive fabric (WooYang Materials, Daegu, Korea) and soft silicone rubber (EcoFlex 0030, Smooth on, Macungie, PA, USA) were used. The fabric was cut using a laser cutter to ensure homogeneous dimensions. Before curing the silicone rubber, the conductive fabric was connected to a thin electric wire (thickness of less than 1 mm) with a single inner coil. The electric wire was stripped and skewered onto a conductive fabric to prevent separation from the fabric. Additionally, electric paint (Bare conductive, London, UK) was spread on the skewed electric wire to guarantee conductivity. Simultaneously, instant glue (LockTite 416, Henkel, Düsseldorf, Germany) was used on the cover of the wire to fix the electric wire to the fabric.

**Figure 3:**
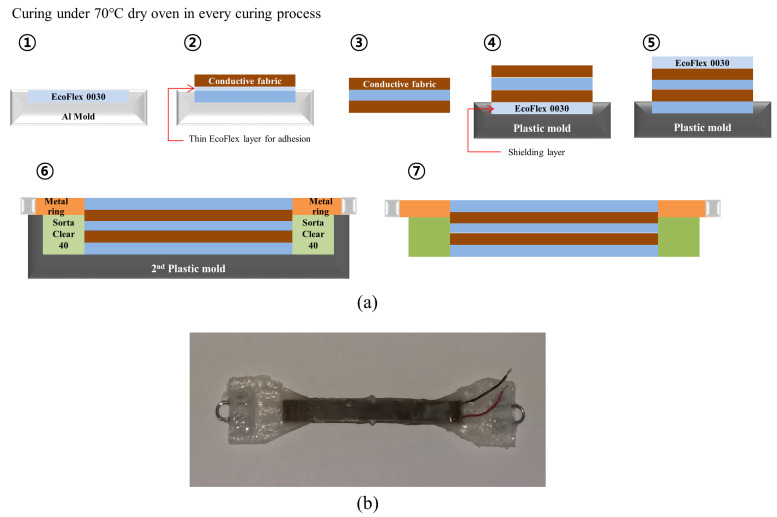
(**a**) Sensor fabrication process. Curing was performed at 70 ℃ for 10 min in drying oven in every curing process. (**b**) Fabricated sensor.

Figure 3a shows the sensor fabrication procedure. To prevent rumpling of the uncured silicone rubber, all fabrication processes were performed with the aluminum mold shown in Figure S1a, and a 3D-printed plastic mold (Figure S1b,c). To cure the silicone layer, EcoFlex 0030 and a hardener were blended in a weight ratio of 1:1. The mixture was poured onto an aluminum mold and degassed in a vacuum chamber for 5 min (0.6–0.8 bar) to fabricate a dielectric layer (① in Figure 3a). The EcoFlex 0030 layer was cured in a dry oven (SH Scientific, Bucheon, Korea) at 70 °C for 10 min. After curing the dielectric layer, a thin adhesion silicone layer was spread on the dielectric layer (② in Figure 3a). The first fabric was attached to the adhesion layer. The cured fabric with a dielectric layer was separated from the aluminum mold and flipped, and the same process was repeated (③ in Figure 3a). Both fabrics were used as the signal and ground layers, respectively. After fabrication, additional insulation layers were cured on the top of and under the sensor on another mold, as shown in Figure S1b. The bottom insulation layer was cured using the same process as that used for the dielectric layer. The fabricated sensor was attached to the insulation layer (④ in Figure 3a). Finally, the top insulation layer was cured (⑤ in Figure 3a), and the sensor was removed from the mold. After the insulation layer hardened, the flexible sensor was removed from the mold and moved (⑥ of Figure 3a) to another mold (Figure S1c) for curing with hard silicone rubber (Sorta Clear 40, Smooth on, Macungie, PA, USA). This part connects the sensor to the ankle brace with a small metal ring and is also useful for the pre-tension of the flexible sensor. The metal ring was sewn onto the fabric, and this helped cure the Sorta Clear 40 rubber, which has a higher tensile strength (5.6 MPa) than EcoFlex 0030 (1.4 MPa). The use of Sorta Clear 40 helped ensure pure flexion of the sensor part without flexion of the connecting part. After curing the connection part, the entire sensor was removed from the mold (⑦ in Figure 3a). The fabricated sensor is shown in Figure 3b.

### 2.3. Sensor Dimensions

To evaluate the performance of the sensors with different dimensions, we fabricated sensors with different lengths and thicknesses. The length of the sensor was defined based on the maximum plantarflexion movement range and height from the plantar to the medial malleolus. All information was gathered from the 6th measurement for adults aged between 20 and 59 years [25]. The body size and movement data from the 6th measurement are shown in Table 1.

**Figure 4:**
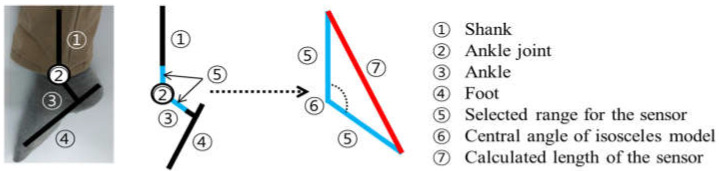
Determination of the isosceles model for determination of the sensor dimensions. Length of the sensor was decided by ⑤ and ⑥.

Using these data, suitable dimensions for the flexible sensors were calculated. Figure 4 shows the theoretical model used to determine the sensor dimensions. The center of rotation was determined to be the medial malleolus. The average height of the medial malleolus was 78.44 mm.

However, this value is from the plantar to the medial malleolus; therefore, we should consider the position of the hooks. To determine the position of hooks, we gathered the average height of the heel point and upper heel point from the 6th Korean body size data (2010) [25]. The values were 22.95 mm and 49.33 mm, respectively. The midpoint between these two points was calculated as approximately 36 mm. An isosceles triangle was drawn from the medial malleolus based on the ankle and shank segments. The center angle of the isosceles triangle was 144.5°, which was measured from the average value of the maximum plantarflexion range of 35.5°. The ends of the isosceles triangle were determined as the positions of the hooks.

Without pre-tension, the sensors can buckle when the ankle is in the maximum plantarflexion position. Buckling causes incorrect signals. To prevent buckling of the sensor, the length of the sensor during maximum plantarflexion was set to 125% of the pre-tensioned length, except for the hard silicone part. Based on the triangle mentioned above, the longest side of the triangle was calculated as 80.7 mm. As the hooks were not flexible and could not function as sensors, the length of the hooks was subtracted. Then, the longest side was reduced to 60.7 mm. Finally, when 25% of the pre-tension was removed, the length was reduced to approximately 49 mm. We considered this value the standard length of the sensor.

**Figure 5:**
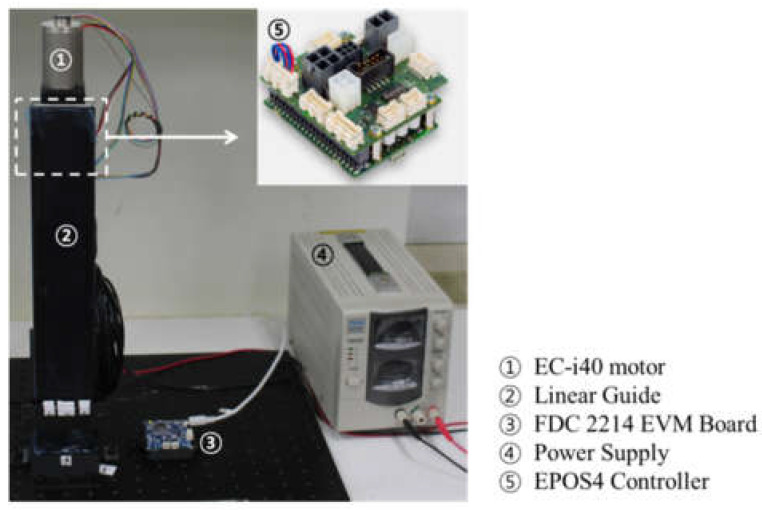
Linear actuator system for the sensor characterization experiment. The motor controller was placed behind the linear guide.

Table 2 shows the dimension of the sensors without additional tension. The width of the sensor was randomly selected for a fixed length of 49 mm. Figure 5 shows the ankle brace (AmiGlobal, Busan, Korea) with a metal hook (upper). The sensors can be applied to the metal hook of the ankle brace by a metal ring at both ends of the sensor (lower).

### 2.4. Sensor Connection and Equipment

After fabrication, the sensor cable was connected to a two-channel Molex 5264 pin. This could then be connected to a customized Arduino board with a Bluetooth module (I2A Systems, Daejeon, Korea). The sensor was equipped with an ankle brace before being connected to the board.

**Figure 6:**
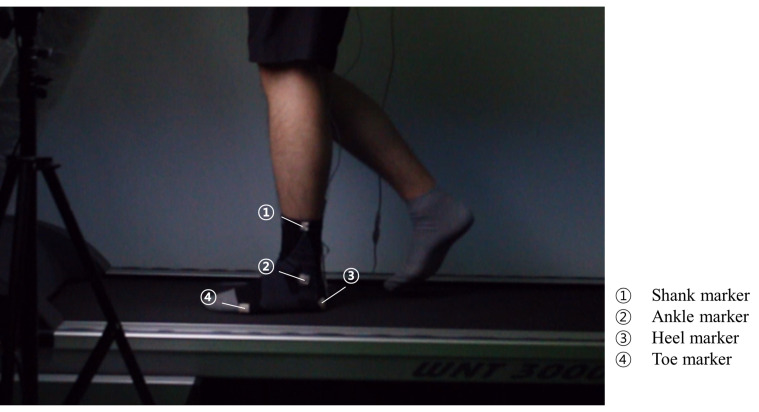
Subject walking on the treadmill with a marked brace.

To guarantee comfortable walking, the MCU board system was worn on the waist (Figure 6). The length of the sensor cable was increased, which helped the subjects walk more comfortably. The sampling rate of the sensor system was set to 50 Hz.

## 3. Experiments

### 3.1. Sensor Characterizations

Repeatability tests were performed to characterize the sensor. In [15,16], the authors characterized their sensor at a speed of 25 mm/s, which is the maximum tension speed of a conventional tension test machine. We decided to use a linear actuation system with the same tension speed as mentioned above for the sensor characterization experiments. During 100 cycles of oscillation, the amplitude of the sensing value was recorded using a capacitance evaluation chip FDC 2214 EVM (Texas Instruments, Texas, USA).

As a subject moved their ankle with a brace and sensor, it was difficult for the sensors to extend to over 150% of their own length; they could be extended to 180% of their own length at a maximum of 30% pre-tension. We decided on a maximum tension range for all the sensors of 150% of their own length in the characterization experiment. A customized linear actuator was used in the experiment. A linear guide (SKR3306C, Samik THK, Suwon, Korea), an EC motor (667306 EC-i40, Maxon Motor, Sachseln, Switzerland) and a motor controller (EPOS4, Maxon Motor, Sachseln, Switzerland) were used for the linear actuator. The linear actuator moved 6 mm in one revolution of the EC motor. Using this ratio, the oscillation range was converted into an incremental encoder unit of the EC motor.

**Figure 7:**
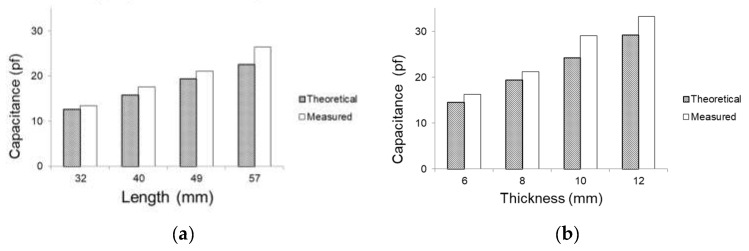
Sensor value comparison between theoretical and measured values. Categorized by the (**a**) length and (**b**) thickness of sensors.

The EC motor was actuated with a sinusoidal profile at a speed of 250 rpm (25 mm/s) with respect to the sensors. The mount for fixing the sensor was printed using a 3D printer (M200, Zortax, Seongnam, Korea). A metal hook was sewed to the mount. Figure 7a shows the linear actuator system. During oscillation, the sensor value was recorded by the evaluation module (FDC 2214 EVM, Texas Instruments, Texas, USA) using the Sensing Solution EVM GUI software. The sampling rate was fixed at 25 Hz. Motor control was programmed using LabView.

### 3.2. Gait Assessment Experiments

Ankle angle measurements were performed on healthy subjects with a healthy gait who had not undergone orthopedic surgery. Experiments were conducted on a treadmill (WNT 3000T, KimSports, Sejong, Korea) with a walking speed of 1.0 m/s (3.6 km/h). This value is based on the minimum velocity range of human walking reported in [16]. Only flat levels were considered in the experiments. Each subject wore an ankle brace and hooked the sensor to the metal ring on the ankle brace. Then, the subject walked for 1 min on the treadmill. The sensor repeated flexion and extension during walking, and its signal was recorded on a sensor board in real time.

At the same time, to measure the real ankle angle during the gait, the gait sequence was filmed using a digital camera (Canon DS 126441, Canon, Tokyo, Japan) with additional light (Compac 408, NanLight, Seoul, Korea). To achieve a clear film without motion blur, the aperture was set to operate 1000 times per second. The distance between the camera and treadmill was 2.4 m. To obtain various gait analysis results, we processed our experiment with four different subjects. Table 3 provides the information for each subject. The subjects were asked to provide visual feedback by dropping a small object at the beginning of each gait cycle. Simultaneously, the moderator started recording the sensor signal. Visual feedback was necessary for movie analysis in Kinovea because all sounds disappeared during the analysis. It was necessary to match both signals, as this allowed us to accurately determine the starting point of the analysis in Kinovea.

**Figure 8:**
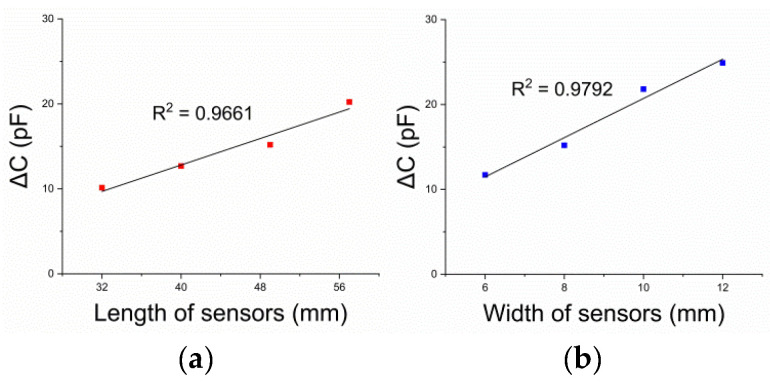
Full-scale output with (**a**) different lengths and (**b**) different thicknesses of each sensor under 50% strain. FSO was represented in (pF) units to compare the change in capacitance with the dimensions of the sensors.

Filmed movies were analyzed in Kinovea, which is widely used in motion tracking research [26,27,28,29]. To synchronize both the sensor signal and the movie, the frame rate of the movie was set to 50 FPS. Low-quality movie files and uneven sensor values were discarded. The subjects wore a marked ankle brace to track the position of each segment. Figure 8 shows a subject walking on a treadmill with a color-marked ankle brace. The marker was attached to the forefoot, heel, ankle, and shank. With the trajectories of these markers, the trajectories of the foot and leg could be calculated. After the angle of each segment was derived, the ankle angle was calculated, as mentioned in [30]. In this study, a positive change denoted dorsiflexion, and a negative change indicated plantar flexion. For gait analysis, we randomly selected three sequential steps from the sensor signal. The same steps were selected for the filmed movie. The selected steps were found in the movie based on the visual effects of the subjects. We then tracked the position of each marker on the ankle brace mentioned above during step sequences. After tracking, we calculated the ankle angle of the selected steps as in [30].

The output from the sensor was converted to a degree unit (°). We measured the value of the sensor at 100% strain. Then, using the output value of each sensor output, we calculated the applied strain for each gait cycle in length units (mm). Finally, using the maximum angle range of each gait, we calculated the ratio between the sensor strain and the ankle angle. The accuracy of the sensor signal is represented as the RMS error between the video and the sensor signal. When the frame rate of the video and the sampling rate of the sensor signal are the same, we can calculate the RMS error.

## 4. Results

### 4.1. Characterizations

**Figure 9:**
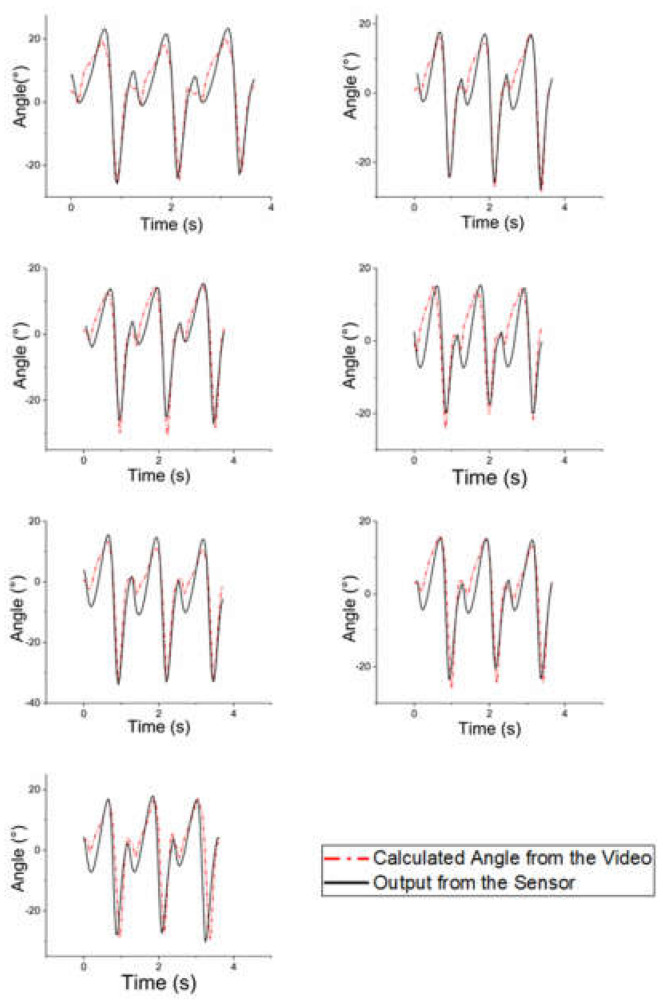
Gait analysis of the first subject. Figures are in the order of the thicknesses (6, 8, 10, 12 mm) and lengths (32, 40, 57 mm) of each sensor.

First, we measured the initial value of each sensor and compared it with the theoretical capacitance value. A digital capacitance meter (LCR-6300, GW Instek, Xinbei, Taiwan) was used to measure the capacitance level of the sensors. The results are presented in Figure 9. The maximum error was 16.95%, which occurred for the sensor with dimensions of 57 × 8 mm. In most cases, the error was less than 10%. Both the measured and theoretical initial capacitance levels of each sensor increased linearly with respect to the dimensions. In Figure 9, the theoretical and measured values are compared in two ways: by the length and thickness of the sensors.

The results of the characterization are shown in Figure S2. During 100 cycles of extension, 20 randomly selected cycles were used for the repetition test. Eventually, on the scale of the relative capacitance, there was no significant difference. Despite the dimensions, from a minimum of 0.7 to a maximum of 0.8, the relative capacitance of the full-scale output (FSO) was measured with respect to width and length variations. No significant change in the relative capacitance value occurred during oscillation.

Hysteresis loops were calculated from 20 randomly selected cycles of repetition, as mentioned above. The maximum error of hysteresis was 21%, which was measured from the sensor with dimensions of 10 × 49 mm. In contrast, the sensor with dimensions of 8 × 57 mm had an 8% hysteresis error, which was the minimum value. The average value of the hysteresis error was 12%.

As predicted, the FSO in the capacitance unit (pF) increased with respect to the sensor dimensions. The $R^{2}$ values for linearity were calculated to be less than 5% for both thickness and length (0.9792 and 0.9661, respectively). For the capacitance, a linearly increasing value was observed with respect to the dimension. Figure 8 shows the variation in the capacitance of the sensors with different lengths and thicknesses.

### 4.2. Gait Experiment Results

In this section, the results of the gait experiment are presented. Figure 9 shows a representative result of the comparison between the sensor signal and the calculated ankle angle of the first subject.

In most cases, the calculated ankle angle (red dotted line) shows a similar form to the trajectory of the ankle angle during walking. In addition, the sensor signals (black line) were fitted to the real angle values. Table 4 presents the mean RMS errors for each sensor. Regardless of its fitness, the RMS error was observed to be at least 3.13°. For the first subject, the sensor with dimensions of 49 × 12 mm recorded a larger RMS error than the other 49 mm-long sensors. For each subject, the smallest RMS was calculated for 49 mm-long sensors with thicknesses of 8 mm or 10 mm. The largest average RMS error was recorded for the fourth subject, while the lowest average RMS error was recorded for the second subject. Owing to the large mismatch between the gait pattern signals from the sensors and the real walking trajectory, a relatively large RMS error was recorded for the third subject despite the subject having the smallest movement range.

## 5. Discussion

The goal of this study was to determine the differences in the accuracy of gait analysis obtained with flexible capacitance-type sensors of different dimensions. Many previous studies have used flexible sensors to measure human movements [15,16,31]. The results of previous studies indicate that the accuracy of the flexible sensor is reasonable.

In the characterization experiment, all the sensors had a hysteresis error of at least 8%. We observed a hysteresis error of more than 20% for the sensor with dimensions of 10 × 49 mm as the highest error. However, the result of the gait analysis was not significantly influenced by the hysteresis error. Moreover, as the purpose of this study was to determine optimized dimensions for the sensor rather than to improve the performance of the sensor, we focused on gait analysis only rather than the hysteresis error. We expect that the results of the gait analysis would have been more reliable if compensation for the hysteresis error had been conducted.

As expected, larger sensors showed a larger capacitance output than smaller sensors with respect to both the length and thickness. In addition, this value had less than 5% $R^{2}$ linearity.

In this study, we did not use any traditional sensors, such as IR cameras or IMUs, to evaluate the output from the sensor. Instead, we filmed all the gait procedures using a digital camera. Then, we randomly selected three sequential steps for comparison with the sensor signal. The selected steps were analyzed in Kinovea, a widely used motion tracking software. Three steps could be recognized as a small part of the entire gait cycle. However, in a previous study [32], the authors conducted a gait analysis comparison experiment with at least three steps. Thus, this was not an unreasonable experimental setup. All the sensors were calibrated before the gait analysis.

In the gait analysis, the gait pattern signals from the sensors were similar to the real walking pattern. However, a mismatch between both signals caused a large RMS error. This mismatch was also observed in a previous study [15]. During plantarflexion, as both signals markedly decreased, a slight mismatch between the two signals was observed. This mismatch caused a larger RMS error than in the other cases. The mismatch cannot be solved by shifting each signal. We hypothesized that it was due to the nonlinear behavior of silicone rubber, as mentioned in [33,34,35]. To overcome this problem, other materials for sensors that can reduce nonlinear behavior should be considered.

Our hypothesis was that the dimensions of the sensor would influence its accuracy. Although the difference was not significant, the lengths of the sensors presented in this study generally had low RMS errors. Regardless of the range of motion, the 49 mm-long sensor tracked real movement with high accuracy, especially for thicknesses of 8 and 10 mm. The accuracy decreased for sensors longer than 49 mm or thicker than 10 mm, as mentioned above. However, the sensors that were shorter than 49 mm did not record an outstanding output compared to the 49 mm-long sensors. The width of the sensor did not have a significant effect in this study if it was not very large. The results of the first subject, who wore a 12 mm-thick sensor and a 57 mm-long sensor, reflect this. This can also be observed in other studies. The largest RMS error was calculated for the sensor with dimensions of 32 × 8 mm. It seems that it is possible to select a width that does not make it difficult to fix the sensor onto the body.

There were two issues with Kinovea: First, the synchronization of both signals was the biggest challenge in this study. We could record the signal of the sensor in real time; however, it could not be used for real angle data because Kinovea analysis was an ex-post process. This caused several mismatches, and some of them could not be fixed by tuning or calibration. The other issue was the blurring of the calculated angle due to the fine error of auto-tracking in Kinovea. This is difficult to control in detail. For instance, as presented in Figure 9, errors occurred mainly from the heel strike to the mid-stance phase. Especially during the heel strike, the markers had relatively small movement compared to the other phases. This caused a relatively small range of foot flat and big error compared to those in the other phases. The use of a high-speed camera would be helpful for calculating a more accurate trajectory. A high-frequency data acquisition (DAQ) system that can chase the frame rate of a high-speed camera should be considered.

During the gait analysis, the sensor was attached to a small metal ring. To equip the sensor, a metal ring should be attached to the ankle brace. However, in real life, attaching a ring to a garment is not a very pleasant option. As mentioned in the first section, a flexible sensor was developed to detect the movement of humans without obstruction, such as area problems, hard materials, or uncomfortable problems. Comfort problems include not only materials but also equipment procedures. Thus, to develop a sensor for real-life analysis, a simpler equipping method should be considered. The use of clips or tongs instead of metal rings may be helpful.

## 6. Conclusions

In this study, we developed a wearable flexible sensor for gait detection. This flexible sensor can be worn more easily than existing devices and does not require a large area. To achieve a higher accuracy, we established a decision on the dimensions of the sensor by considering the average body size and range of motion of adults aged between 20 and 59 years. We characterized all the sensors and achieved a hysteresis error of at least 8%. The initial value of the sensor did not significantly differ from its theoretical value.

The sensor detects changes in the electricity capacitance that occur when it contracts and extends. When the sensor is used to measure the ankle angle, it repeatedly undergoes flexion and relaxation owing to the movement of the ankle. A brace-type sensor system can therefore record the movement of the ankle while walking. In the gait analysis, the ankle angle calculated from Kinovea was compared with the sensor signal. Gait analysis was conducted under the same conditions for all the subjects in this study. During gait analysis, the sensor recorded a minimum RMS error of 3.13°. This result was obtained with the standard dimensions proposed in this study. The results reveal that the factor that most affects the accuracy of the sensor is its length. In addition, excessively large dimensions can interfere with the accuracy of the sensor.

In future studies, we will focus on multi-dimensional gait analysis. A gait cannot be expressed as a one-dimensional motion because of the structure of the ankle. Moreover, according to clinical data, patients with drop foot have pathological gait patterns in both dorsiflexion/plantarflexion and abduction/adduction. Thus, we will use an additional sensor to measure ankle abduction and adduction during walking. In addition, to overcome the nonlinear behavior of the sensor, changes in the main material or composition are also worth considering.

## Figures and Tables

**Table 1:** Average body size of adults aged between 20 and 59 [25].

Properties	Value
Medial malleolus height	78.44 mm
Maximum plantarflexion	35.5°
Higher heel height	49.33 mm
Heel height	22.95 mm

**Table 2:** Dimensions of the sensors.

Properties	Value
Length	32 mm
	40 mm
	49 mm
	57 mm
Width	6 mm
	8 mm
	10 mm
	12 mm

**Table 3:** Profiles of the subjects.

Sex	Age	Height (cm)	Body Mass (kg)
M	32	171 cm	61.3 kg
M	28	166 cm	73.9 kg
M	31	174 cm	80.2 kg
M	27	167 cm	80.4 kg

**Table 4:** RMS errors of each sensor from the results for each subject.

Dimension	RMS Error (°)			
	1st Subject	2nd Subject	3rd Subject	4th Subject
49 × 6 mm	3.92	3.42	4.79	5.06
49 × 8 mm	3.76	3.13	4.54	4.75
49 × 10 mm	3.47	3.41	4.47	4.29
49 × 12 mm	6.11	4.18	4.55	4.89
32 × 8 mm	4.81	3.69	4.80	4.87
40 × 8 mm	4.53	3.50	5.23	5.95
57 × 8 mm	7.98	3.99	5.31	5.47

## Data Availability

The data are not publicly available due to privacy restriction.

## Supplementary Materials

The following are available online at https://www.mdpi.com/article/10.3390/s21165299/s1, Figure S1: (**a**) CAD image of molds for dielectric layer curing. All molds have a depth of 0.5 mm to accommodate the thickness of the dielectric layer. (**b**) CAD image of plastic molds for protection layer with connector for linkage. (**c**) CAD image of plastic molds for curing the linkage. Linkage consisted of Sorta Clear 40 and a small metal ring with fabric, Figure S2: Hysteresis loop of each sensor during 20 cycles of repetitious stretching. In order, these represent 6 mm, 8 mm, 10 mm, 12 mm (thickness), 32 mm, 40 mm, and 57 mm (length), Figure S3: Gait analysis of 2nd subject. Figures are in order by thickness (6, 8, 10, 12 mm) and length (32, 40, 57 mm) of each sensor, Figure S4: Gait analysis of 3rd subject. Figures are in order by thickness (6, 8, 10, 12 mm) and length (32, 40, 57 mm) of each sensor, Figure S5: Gait analysis of 4th subject. Figures are in order by thickness (6, 8, 10, 12 mm) and length (32, 40, 57 mm) of each sensor, Figure S6: Example of one gait cycle that sorted by gait cycle percentile and gait events. (a) Heel strike, (b) foot flat, (c) heel off, (d) toe off and (e) heel strike of next step. This graph was from the 1st subject with 8 mm × 49 mm dimension sensor, Table S1: 25th, 50th, 75th and 95th quartile of heel height and higher heel height from 6th Korean body size measurement. These values were used to calculate 32, 40 and 57 mm length.
