# Supplementary material for: Gait Analysis Accuracy Difference with Different Dimensions of Flexible Capacitance Sensors
Source: Sensors (Basel). 2021 Aug 5;21(16):5299. doi: 10.3390/s21165299 (PMC8401030; doi:10.3390/s21165299)
Supplement: Supplementary file 1 [file sensors-21-05299-s001.zip › sensors-1283518-supplementary.pdf]

sensors

MDPI

Supplementary materials

Gait Analysis Accuracy Difference with Different Dimensions of Flexible Capacitance Sensors

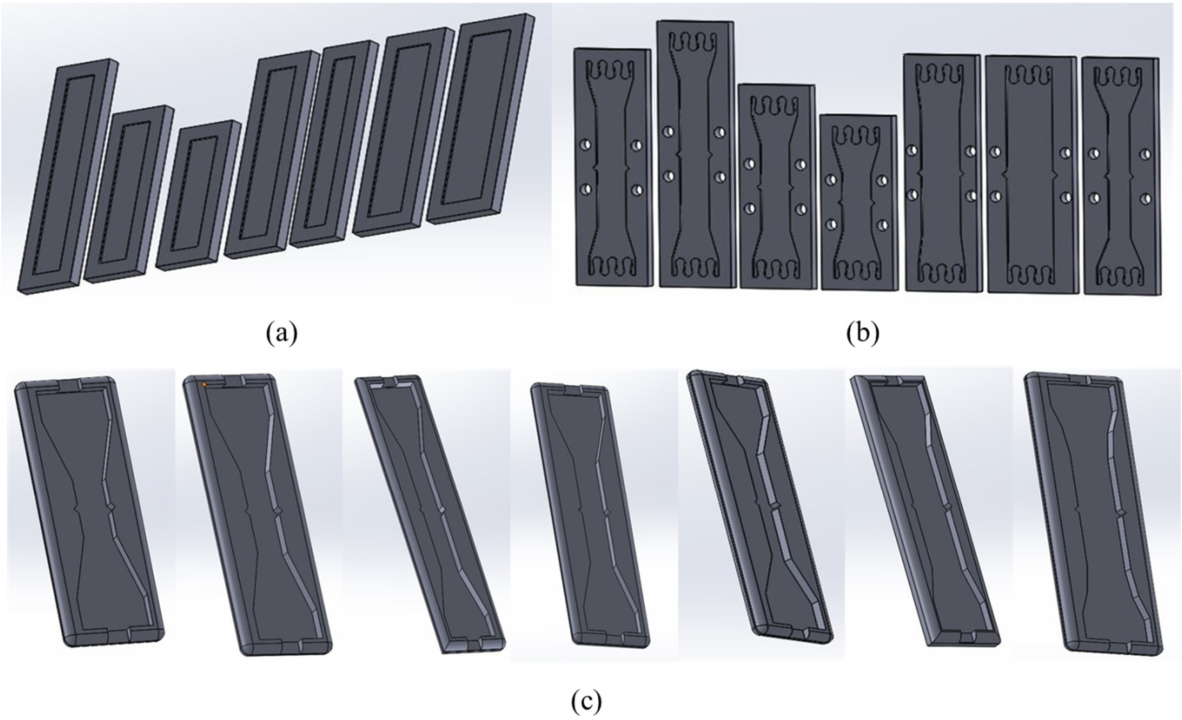

Figure S1. (**a**) CAD image of molds for dielectric layer curing. All molds have a depth of 0.5 mm to accommodate the thickness of the dielectric layer. (**b**) CAD image of plastic molds for protection layer with connector for linkage. (**c**) CAD image of plastic molds for curing the linkage. Linkage consisted of Sorta Clear 40 and a small metal ring with fabric.

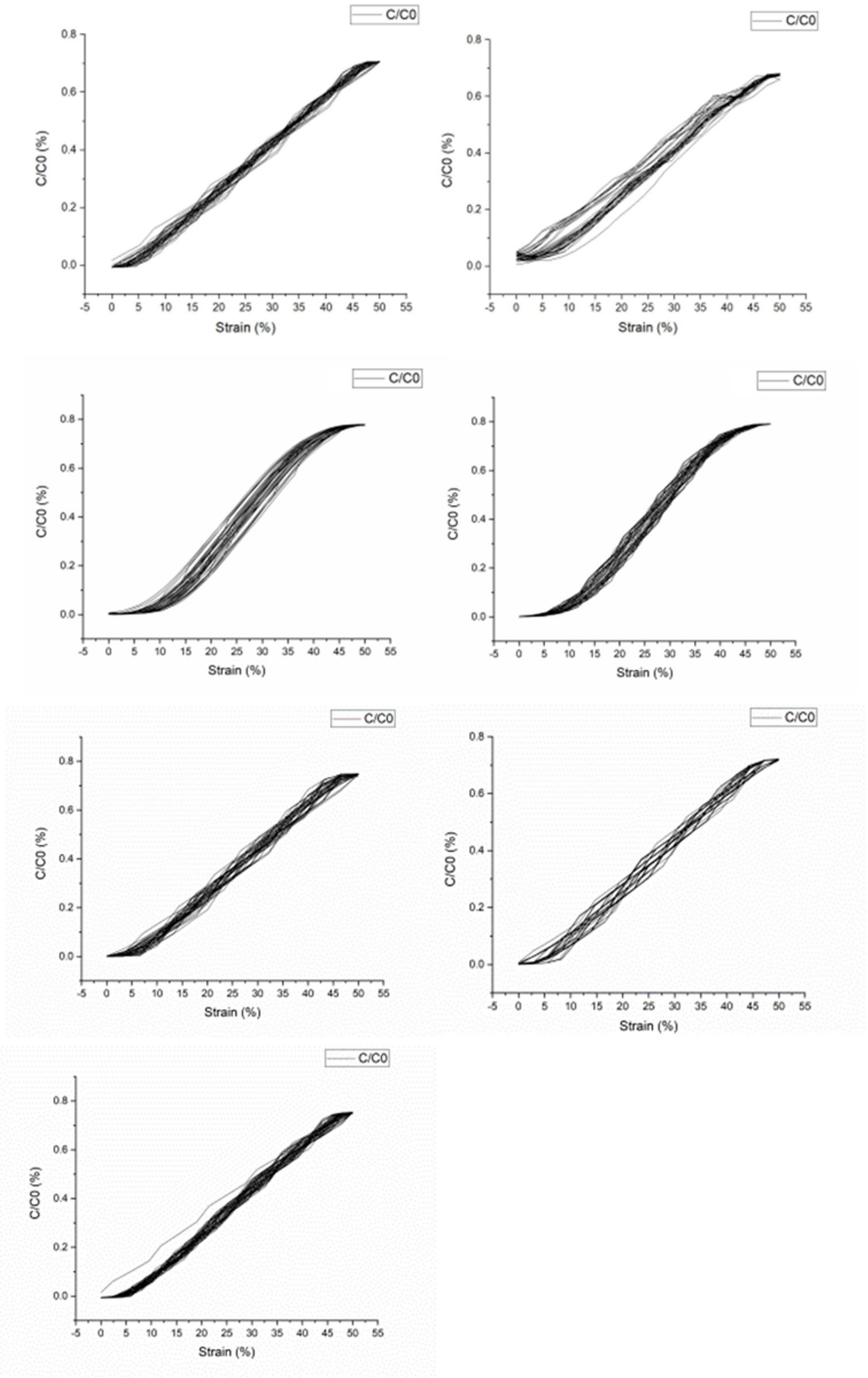

Figure S2. Hysteresis loop of each sensor during 20 cycles of repetitious stretching. In order, these represent 6 mm, 8 mm, 10 mm, 12 mm (thickness), 32 mm, 40 mm, and 57 mm (length).

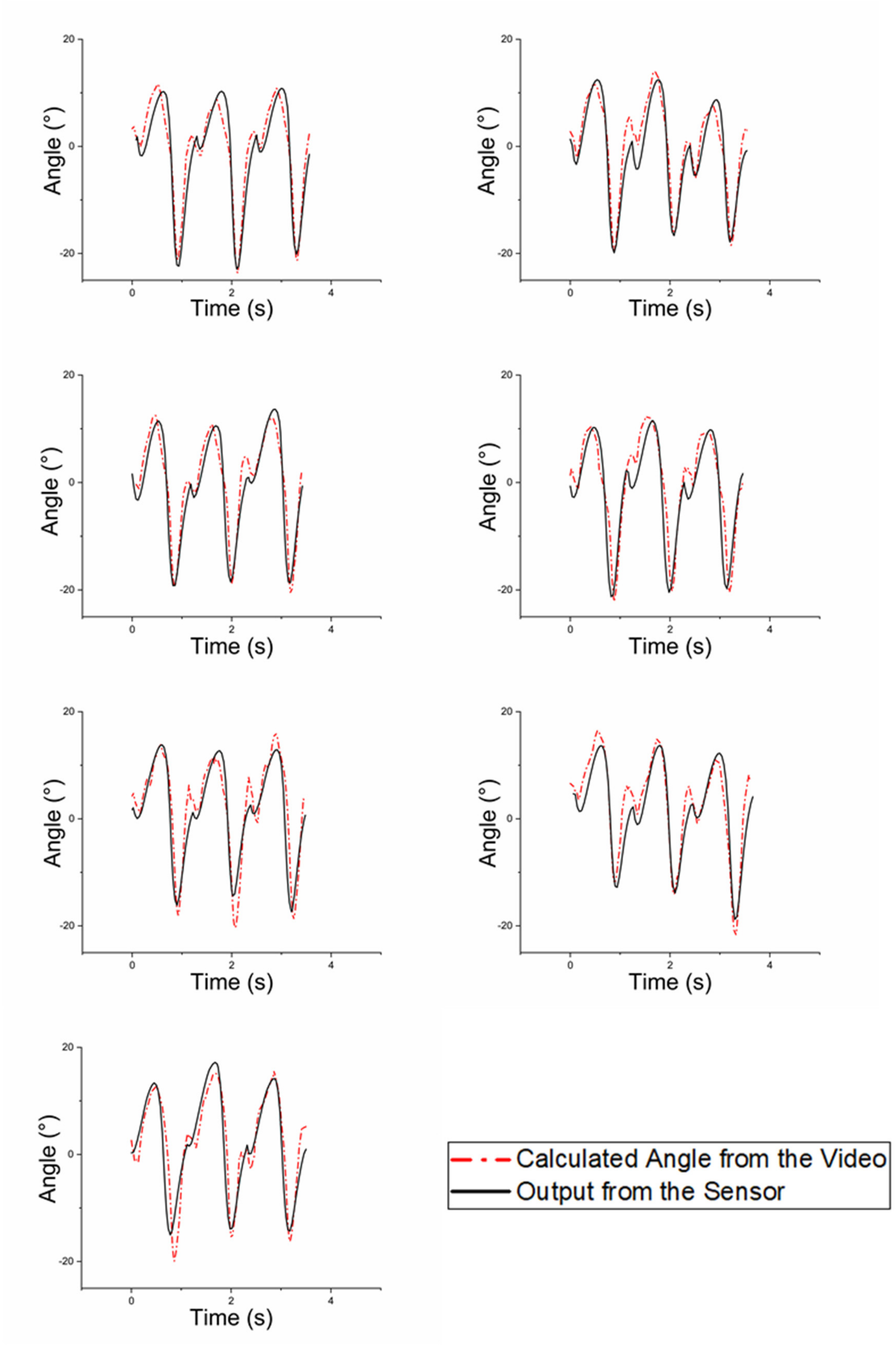

Figure S3. Gait analysis of 2nd subject. Figures are in order by thickness (6, 8, 10, 12 mm) and length (32, 40, 57 mm) of each sensor.

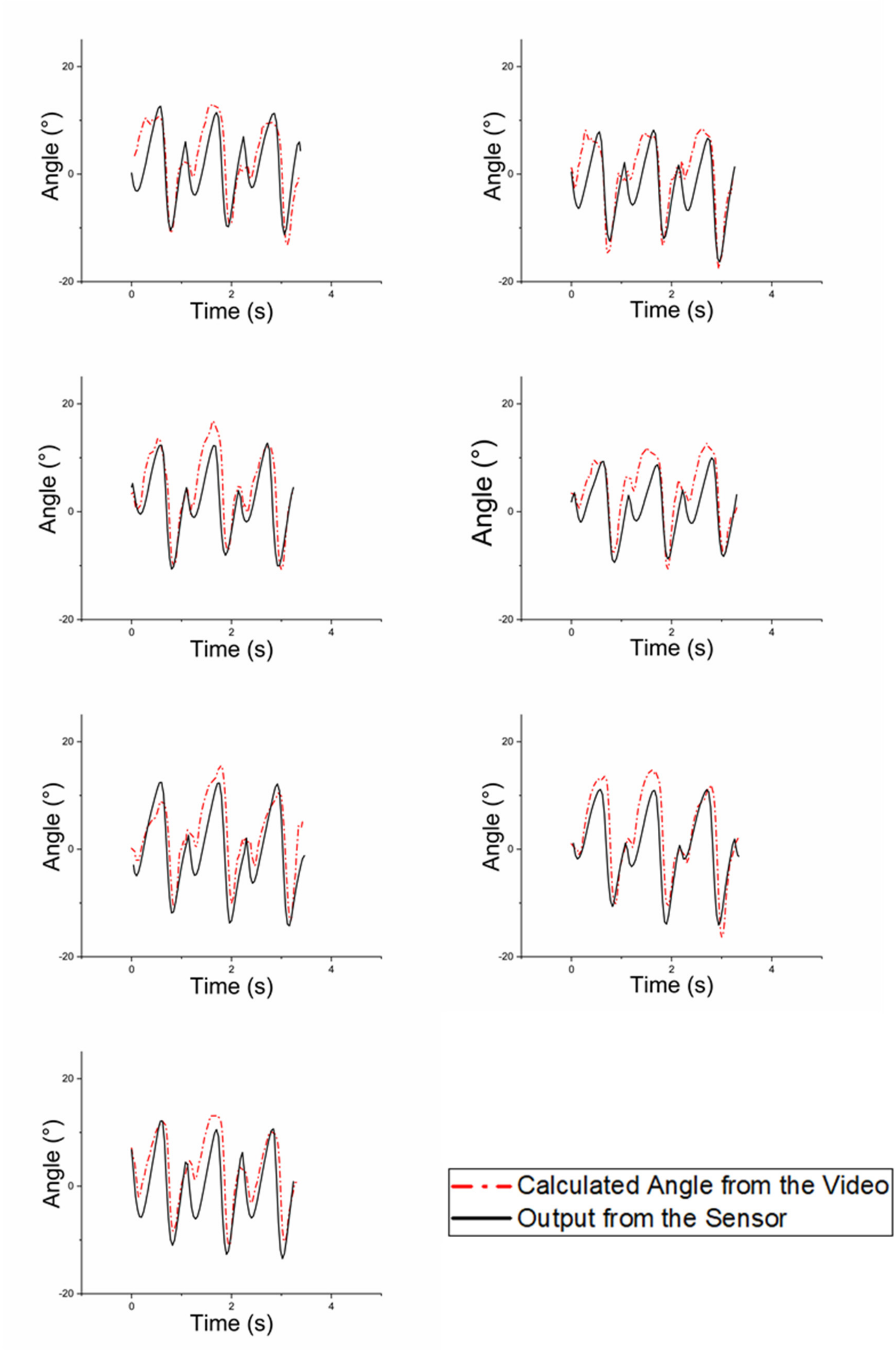

Figure S4. Gait analysis of 3rd subject. Figures are in order by thickness (6, 8, 10, 12 mm) and length (32, 40, 57 mm) of each sensor.

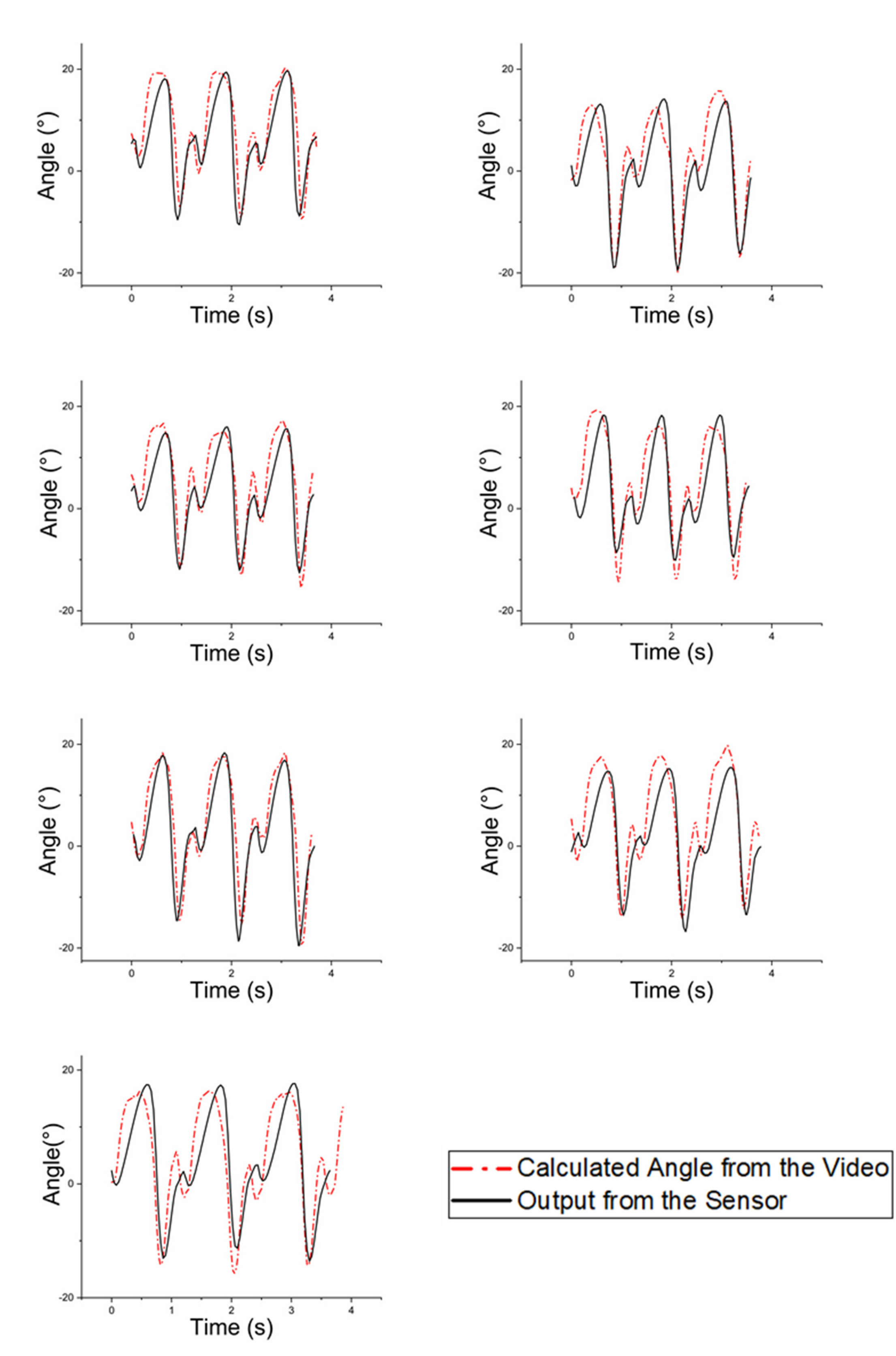

Figure S5. Gait analysis of 4th subject. Figures are in order by thickness (6, 8, 10, 12 mm) and length (32, 40, 57 mm) of each sensor.

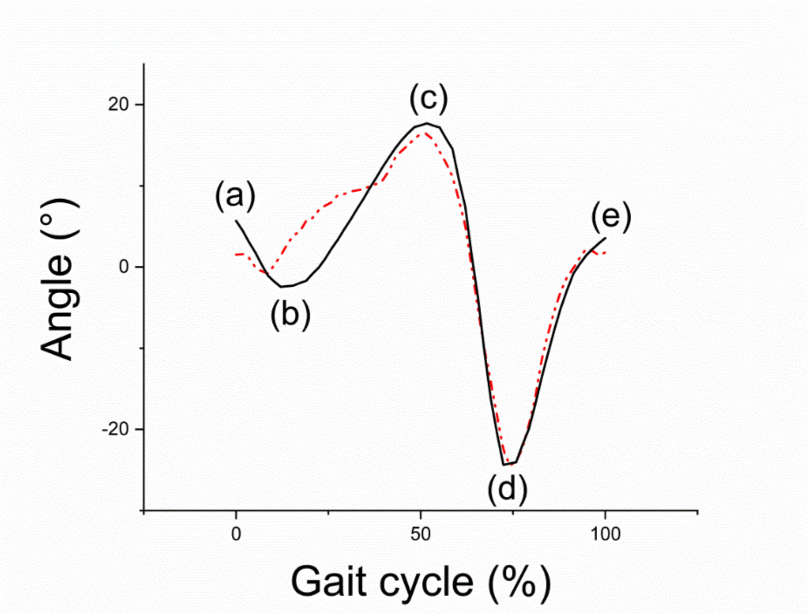

Figure S6. Example of one gait cycle that sorted by gait cycle percentile and gait events. (a) Heel strike, (b) foot flat, (c) heel off, (d) toe off and (e) heel strike of next step. This graph was from the 1st subject with 8 mm x 49 mm dimension sensor.

Table S1. 25th, 50th, 75th and 95th quartile of heel height and higher heel height from 6th Korean body size measurement. These values were used to calculate 32, 40 and 57 mm length.

**Body size**	**Quartile**			
	**25th**	**50th**	**75th**	**95th**
Heel height	19.85 mm	22.85 mm	25.95 mm	29.70 mm
Higher heel height	39.75 mm	47.95 mm	58.10 mm	67.05 mm
